# Ultrastructure of hyphal cells of *Trichophyton tonsurans*

**DOI:** 10.18502/cmm.6.1.2508

**Published:** 2020

**Authors:** Amaliya Stepanova, G. Sybren de Hoog, Nataliya Vasilyeva, Konstantin Raznatovskiy, Galina Chilina

**Affiliations:** 1 North-Western State Medical University named after I.I. Mechnikov: Kashkin Research Institute of Medical Mycology, Saint Petersburg, Russia; 2 Westerdijk Fungal Biodiversity Institute, Utrecht, The Netherlands; 3 Centre of Expertise in Mycology of Radboud University Medical Centre / Canisius Wilhelmina Hospital, Nijmegen, The Netherlands; 4 Department of Dermatovenerology, North-Western State Medical University I.I. Mechnikov, St. Petersburg, Russia

**Keywords:** Dermatophytes, Morphogenesis, Septal pore apparatus, Ultrastructure

## Abstract

**Background and Purpose:**

*Trichophyton tonsurans *is a widely distributed anthropophilic dermatophyte causing different diseases of skin. In the literature limited data are available about the morphogenesis of vegetative mycelium of *T. tonsurans* and related anthropophilic dermatophytes. The aim of present study was to describe ultrastructural patterns of development, cellular organellography and septal pore apparatus structure of *in vitro* growing vegetative mycelium of *T. tonsurans*.

**Materials and Methods:**

*Trichophyton tonsurans* strain RCPFF 214/898 was grown on solid Czapek’s Agar (CzA) at 28ºС. For investigation of colonies morphology we used methods of light-, scanning and transmission electron microscopy (SEM and TEM).

**Results:**

Differences in morphogenesis of aerial and substrate hyphae were revealed. Mitochondrial reticulum and fibrosinous bodies were shown in *T. tonsurans* for the first time. The septal pore apparatus in hyphal cells of was comprised Woronin bodies and septal pore plugs. Woronin bodies (0.18 µm), located with 1‒4 near the pore, were spherical, membrane-bound, and had a homogeneous, electron-dense content. The cells of aerial and submerged hyphal cells of *T. tonsurans *contain two nuclei.

**Conclusion:**

Mature cells of substrate hyphae appeared more active than comparable cells in the aerial mycelium. During the maturation process, the differences in number and morphology of mitochondria, number of vacuoles, and in the synthesis of different types of storage substances were revealed. Presence of “mitochondrial reticulum” and variable types of storage substances in submerged hyphal cells suggested higher levels of metabolic activity compared to aerial mycelium.

## Introduction

*Trichophyton tonsurans *is a widely distributed anthropophilic dermatophyte causing scalp infection (tinea capitis) with endothrix involvement of hair [1]. The disorder frequently occurs in children [2] and adult women [3], and is often transmitted by physical contact, for example during wrestling [4]. Less often the mycosis affects smooth skin, feet or nails [5]. The species has an evolutionary origin from *T. equinum* occurring on horse skin. Kandemir et al. [6] noted that the two species are difficult to distinguish, as they possibly were in a stage of incomplete lineage sorting.

Data about morphogenesis of growing dermatophyte hyphal cells and their organellography are limited [7‒13]. For understanding dermatophyte development, feeding, transport in host tissue, and mechanism of response to antifungal stress, data on cellular organellography and patterns of interactions with host cells are required, including *in vitro *baseline studies of cell behavior and ultrastructure. Structure of septa and septal pore apparatus have great significance to the analysis of cell communication, and ultrastructural data have been applied in fungal diagnostics and phylogeny. Patterns of morphogenesis of vegetative mycelium of *T. tonsurans* and related anthropophilic dermatophytes have not been investigated. Studies were limited to scanning electron microscopic ultrastructure of microconidia [14] and of walls of hyphal cells and chlamydospore‐like cells [15]. The present study focuses on the ultrastructural patterns of hyphal cell morphogenesis, cellular organellography and septal pore apparatus structure of *in vitro* growing vegetative mycelium of *Trichophyton tonsurans*.

## Materials and Methods

*Trichophyton tonsurans* strain RCPFF 214/898, isolated from a human patient with onychomycosis, was obtained from the Russian Collection of Pathogenic Fungi, St. Petersburg (Russia). Identity was confirmed by sequencing the rDNA ITS locus. The strain was grown on solid CzA at 28ºС and investigated after 5, 10, 20 and 30 days of cultivation. Colonies were photographed with an Olympus BX 51 camera. For scanning electron microscopy (SEM), small parts of fungal colonies were fixed in 3% glutaraldehyde (in cacodylate buffer, pH 7.2) for 3 h, post-fixed overnight in 1% osmium tetroxide in the same buffer, dehydrated by ethanol series (30%→50%→70%), critical-point dried (HCP-2) for 15 min, coated with gold and observed in a JSM 35 scanning electron microscope (Jeol, Tokyo, Japan). 

For transmission electron microscopy (TEM), blocks of nutrient medium with parts of fungal colonies were fixed during the 3 h in 3% glutaraldehyde and post-fixed for 10 h in 1% osmium tetroxide. Subsequently, samples were dehydrated through an ethanol and acetone series and embedded in epon-araldite epoxy resin. Ultrathin sections were cut with an Ultratome 2088 (LKB, Bromma, Sweden), stained with uranyl acetate and lead citrate and were investigated under a TEM Jem 100 SX (Jeol). 

## Results

**Figure 1:**
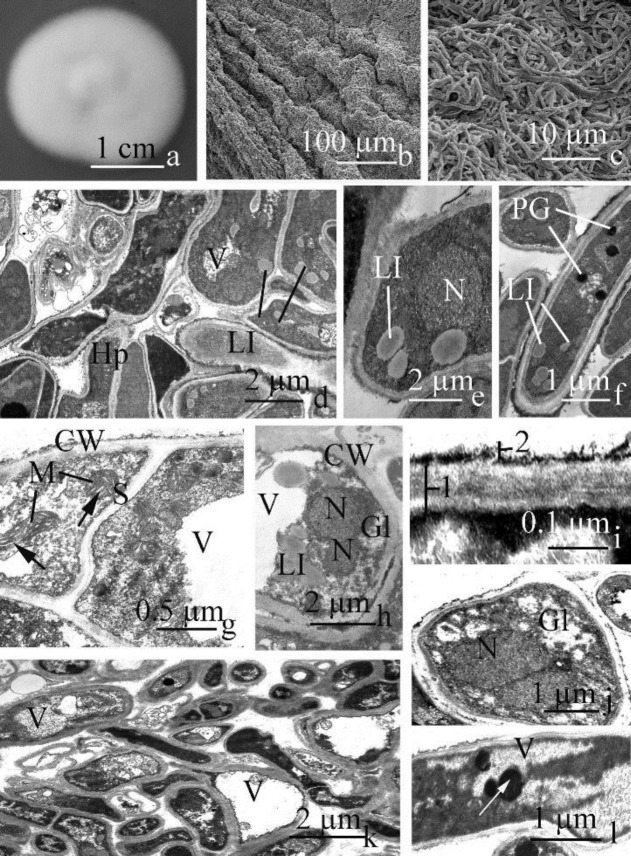
*Trichophyton tonsurans *(RCPFF 214/898). a. Colony after 15 days of cultivation on Czapek’s medium; b, c. Parts of fungal colony under SEM; d-i. Fragments of aerial hyphal cells under TEM; j-l. Fragments of the submerged hyphal cells under TEM

Colonies were slow-growing, reaching a diameter of 3.5 cm after 15 days of incubation on CzA at 28ºC, were raised at the centre and flat near the margin (Figure 1 a), velvety, white, and had a yellowish-brown reverse. 

***Aerial hyphae***

At low magnification observed with SEM, radial development of the colony is visible (Figure 1 b). Young hyphal cells of aerial and submerged mycelial cells contained two adjacent, ellipsoidal interphase nuclei (Figures 1 e, h,) and uniformly distributed small vacuoles (Figure 1 d) which contain thin-fibrillar material. Mitochondria (from 6 to 8 on median cell section) were located at the periphery of cells near the cell wall. They were single or arranged in small groups, spherical (0.6 µm) to ellipsoidal (0.5 × 0.6 µm). The mitochondrial matrix was dark in comparison with cytosol. 

Mature cells of the aerial mycelium varied 2.0‒2.6 µm in width, and were densely compacted (Figures 1 c, d). The two interphase nuclei per cell (Figure 1 j) were ellipsoidal (1.3 × 1.5 µm) with slightly irregular nuclear envelopes. A small (0.3 µm), dark nucleolus was localized near nuclear envelope. The electron-density of the nucleoplasm was similar to that of the cytosol. Moderate amounts of condensed chromatin were distributed in the nucleoplasm of the interphase nuclei. 

Morphogenesis of cells of the aerial mycelium initiated with the formation of a small number (4‒5 per cell section) of small (0.3‒0.5 µm), single, polymorphic vacuoles (Figure 1 f) which were uniformly distributed in the median cell section. Membranous fragments were observed in the vacuolar contents having variable size and shape, in addition to an aggregation of fibrillar and granular materials with variable electron density, and small (0.2‒0.3 µm), dark protein globules localized near the tonoplast (Figure 1 f). The cytosol contained storage compounds in the form of a moderate number (6‒7 per cell section) of small (0.2‒0.4 µm) median electron-dense lipid inclusions (Figure 1 f) surrounded by typical light peripheral rims.

During subsequent growth of the aerial mycelium, the number of mitochondria per cell did not change significantly. Mitochondria were single, rather small (0.3‒0.5 µm), polymorphic. The mitochondrial matrix was median electron-dense and contained numerous light cristae with various extensions differing in orientation (Figure 1 g, arrows). 

Morphogenesis of aerial cells continued with synthesis of storage lipids and rosettes of glycogen in cytosol and protein globules in vacuoles (Figures 1 h). The lipid inclusions (5‒7 per cell section) in the globules were small (0.2‒0.3 µm), polymorphic, median electron-dense, localized near interphase nuclei and the cell wall. The rosettes of glycogen (0.10‒0.12 µm) having low electron-density often formed small aggregations and occupied a peripheral position in the hyphal cells (Figure 1 h), in close affinity to interphase nucleus and storage lipid inclusions. In mature hyphal cells, storage compounds were the main components of the cytosol, with a dominance of glycogen rosettes. The cytosol had moderate electron-density and contained numerous free ribosomes. Plasma membranes were flat and in close contact with the thin (0.10‒0.13 µm) cell wall. The walls were 2-layered (Figure 1 i), with a thick (0.08‒0.11 µm, Figure 1 i, 1), moderately electron-dense internal and a thin (0.02‒0.03 µm, Figure 1 i), dark, loose, often interrupted external layer with an irregular contour.

***Submerged hyphae***

The cells of a submerged mycelium were also oriented randomly and were densely interwoven (Figure 1 k). Density of hyphal cell distribution was increased during colony growth and differentiation. In the central part of hyphal cells one or two (Figures 1 j) interphase nuclei, that were spherical (1.3 µm diam), ellipsoidal (0.6 × 1.0 µm) or slightly irregular in shape (1.5 µm diam), were located. The condensed chromatin was in moderate amount and randomly distributed upon sectioning of the nucleus. The nucleolus was single, large (0.5 µm diam), localized near the nucleolar envelope, electron-dense, compacted, granular, with the irregular contour. 

In young hyphal cells, small, variously shaped vacuoles fused with one another, leading to larger vacuoles, in which fibrillar and dark, homogeneous material accumulated, which were fragments of concentrically oriented membranes and dark protein globules (Figure 1 l, arrow). In mature hyphal cells, the formation of the central vacuole coincided with cellular transition to a stage of senescence. During maturation, the number of mitochondria increased from 5 to 12 upon median cell section. They were large (0.5‒0.7 µm), polymorphic, with dense dark cristae and a moderately electron-dense matrix. Often elongate mitochondria were revealed in cells of the substrate mycelium what demonstrated the presence of one large organelle the “mitochondrial reticulum” (Figure 3 a), which presence correlated a high level of metabolism. 

Abbreviations used: CW – cell wall, ER – endoplasmic reticulum, FB – fibrosinous body, Gl – glycogen, Hp – hypha, LI – lipid(s) inclusion(s), M – mitochondrium, N – nucleus, Nu – nucleolus, PG – protein globule in vacuole, Pg – plug, S – septum, SC – senescent cell, V – vacuole, WB – Woronin bodies

**Figure 2:**
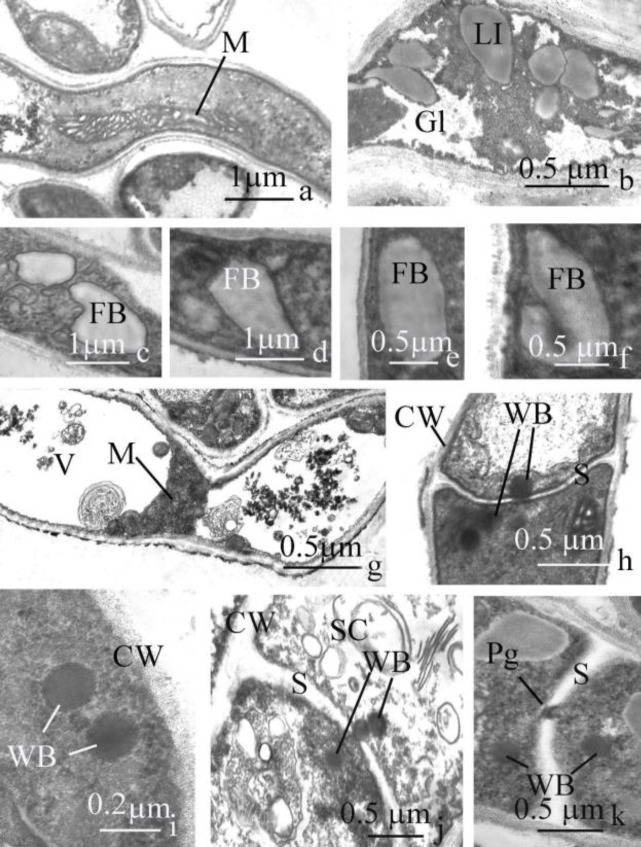
Ultrastructure of *T. tonsurans *(RCPFF 214/898) hyphal cells of submerged mycelium, growing *in vitro*

Short, poorly curved cisterns of smooth endoplasmic reticulum were revealed during the entire period of hyphal cell development. Maturation of cells of substrate hyphae was followed by synthesis of a large number of small (0.10‒0.12 µm), low electronic-dense rosettes of glycogen (Figure 2 b), fibrosinous bodies (Figures 2 c‒f) and lipid inclusions (Figure 2 b). The number of fibrosinous bodies in cytoplasm of hyphal cells varied from 2 to 5. This type of storage substances, as a rule, was single or in small (2‒3) groups, localized near cell walls. Fibrosinous bodies were moderately electron-dense, variable in size (0.5‒2.0 µm) and shape (ellipsoidal, triangular, conical, polygonal, irregular or V-shaped). Polymorphic (0.3‒0.6 µm) lipid inclusions with moderate electron-density were arranged singly or in small groups. 

Among all types of storage compounds observed, the glycogen rosettes were dominant. Cytosol had high electron-density and contained numerous free ribosomes. The plasma membrane was straight or slightly undulate. Cell walls of hyphae of aerial mycelium differed from those of substrate hyphae.

With increasing age of colonies, the frequency of senescent and dead cells in aerial and submerged mycelium progressively increased in a direction from the center to its periphery. Within about 30 days of incubation, living aerial and substrate hyphal cells were practically absent. The final stages of the morphogenesis of cells of a mycelium passed according to identical pattern. Sizes of nucleus and nucleolus decreased about 50%, while the level of vacuolization increased considerably (Figure 2 g) and the amount of storage compounds was reduced. The electron-density of cytosol, as well as the total number of organelles decreased. 

***Septal pore apparatus***

Cells of aerial and submerged hyphae were separated from each other by single-layered, wedge-shaped septa (Figures 2 h, j, k), with thickness of 0.12 µm on average. In the central part of septa, a pore of 0.07 µm wide was observed. Typically, 1-4 spherical Woronin bodies (0.18 µm; Figures 2 h, j) were present. Contents of Woronin bodies were homogeneously electron-dense, while towards the periphery they were surrounded by a high electronic-dense, three-layered membrane. In young hyphal cells, Woronin bodies were sometimes located in cytosol near the cell wall (Figure 2 i). Upon senescence of the adjacent hyphal cell, Woronin bodies occluded the septal pore (Figure 2 j). Another component of the septal pore apparatus was a small, spherical, electron-dense plug (Figure 2 k), which was generally present inside the septal pores of senescent cells of vegetative mycelium.

## Discussion

Our data show that mature aerial and submerged hyphal cells of *T. tonsurans* are similar in number (2 nuclei) and size with respect to the interphase nuclei. In comparison, the hyphal cells of *T. violaceum* are generally multinucleate [7, 8]. The two nuclei were typical for the hyphal cells of *T. rubrum* [9]. Nuclei of *T. tonsurans* submerged hyphal cells were typical variable in shape. During the maturation process, the number of mitochondria increased in hyphal cells of both aerial and submerged mycelium. In hyphal cells of submerged mycelium, mitochondria united together with formation of one giant organelle, termed “mitochondrial reticulum”. This organelle has not been reported for dermatophytes. Main differences between the two hyphal types were revealed in the presence of this mitochondrial reticulum, in the number of vacuoles, and in the synthesis of storage compounds in the form of lipid inclusions, rosettes of glycogen, fibrosinous bodies and dark protein globules in vacuoles. In comparison, rosettes of glycogen and lipid inclusions were revealed in hyphal cells *T. rubrum* [9, 11], rosettes of glycogen, lipid inclusions and dark protein globules in *T. violaceum *[10] and only lipid inclusions in *Trichophyton interdigitale *[13].

Submerged mycelium of* T. tonsurans, *in contrast to aerial hyphae, contain very limited storage compounds such as fibrosinous bodies, which present the polysaccharide storage type [16]. Previously, these bodies were described in the paraphysis-like cells of *Puccinia coronata* f. sp. *avenae* [17], pedicel cells of the uredium of *Melampsora lini* [18], conidia and sometimes in the conidiogenous cells of five species of the powdery mildews *Erysiphe communis*, *Microshaera alphitoides*, *Sphaerotheca pannosa*, *S. fulgineae*, and *S. mors-uvae* [16], hyphal cells of submerged mycelium of *Aspergillus versicolor* [19] and *A. candidus* [20], in *in vitro* growing strains [21], and in the yeast cells of *Cryptococcus neoformans *in human brain [22].

In general, mature cells of growing submerged mycelium had a more active appearance in comparison to aerial hyphae. Possibly this difference is explained by the different roles of submerged and aerial hyphae, i.e. in feeding and sporulation, respectively. Judging from TEM data, the *in vitro* grown hyphal cells of aerial and submerged mycelium of *T. tonsurans* differ from each other in the early patterns of morphogenesis. This was also observed in the dermatophytes *T. rubrum* [9], *T. violaceum* [10] and *T.* *interdigitale *[13]. 

*Trichophyton,* in contrast to the genus *Aspergillus* where Woronin bodies of analyzed members were found to be rather homogeneous in the size and structure [19, 20, 23], was characterized by presence of Woronin bodies with more variable number and morphology. In *T. tonsurans,* typically 1‒4 (0.18 µm), homogeneously electron-dense Woronin bodies were present, in *T.* *interdigitale* 3‒5 (0.08 µm) crystalline bodies with moderate electron density [13], and in *T. rubrum *1‒7 (0.17 µm) homogeneously electron-dense bodies [9]. In investigated species of dermatophytes, Woronin bodies were surrounded by a highly electronic-dense, three-layered membrane. In comparison, in the septal pore apparatus of *Scedosporium apiospermum* and *S. boydii, *crystalline satellites [24] were not surrounded by a membrane.

The ultrastructure of the septal pore apparatus generally is highly conserved between members of a single genus, and is usually applied as a character set at the ordinal level [1, 24]. Only in the basidiomycetous yeast genus *Trichosporon* diverse types of septal pore apparatus are known [25]. Anthropophilic dermatophytes are known to be phylogenetically close, and the genus *Trichophyton* has recently been revised with rearrangement of remote species into *Arthroderma* [1]. The revealed differences between the investigated species of *Trichophyton* are thus far unexplained. Note that electron-dense plugs with the same morphology as in *T. tonsurans* were revealed in the septal pore of old hyphal cells another species of dermatophytes [7‒13].

In mature hyphal cells of *T. tonsurans*, typically a 2-layered outer cell wall (thin outer electron-dense and internal thick moderately electron-dense) is present. A similar structure was described for hyphal cells other species of dermatophytes [7‒13].

## Conclusion

The hyphal cells of *T. tonsurans* contain two nuclei, with variable shape in submerged hyphal cells, which may be related with high metabolic activity. Mature cells of substrate hyphae appeared more active than comparable cells in the aerial mycelium. During the maturation process, the differences in number and morphology of mitochondria, number of vacuoles, and in synthesis of different types of storage substances were revealed. Presence of “mitochondrial reticulum” and variable types of storage substances in the hyphal cells of submerged mycelium suggested about higher levels of metabolic activity in comparison with the aerial mycelium. The septal pore apparatus of *T. tonsurans *hyphal cells typical contains 1‒4 small (0.18 µm) dark Woronin bodies.

## References

[1] De Hoog GS, Guarro J, Gené J, Figueras MJ (2013). Atlas of clinical fungi.

[2] Gupta AK, Mays RR, Versteeg SG, Piraccini BM, Shear NM, Piguet V (2018). Tinea capitis in children: a systematic review of management. J Eur Acad Dermatol Venereol.

[3] Gupta AK, Summerbell RC (1998). Increased incidence of Trichophyton tonsurans tinea capitis in Ontario, Canada between 1985 and 1996. Med Mycol.

[4] el Fari M, Gräser Y, Presber W, Tietz HJ (2000). An epidemic of tinea corporis caused by Trichophyton tonsurans among children (wrestlers) in Germany. Mycoses.

[5] Rasnatovskiy KI, Rodionov АN, Kotrechova LP (2006). Dermatomycoses. The guide for doctors. Moscow.

[6] Kandemir H, Dukik K, Hagen F, Ilkit M, Gräser Y, de Hoog GS (2019). Polyphasic discrimination of Trichophyton tonsurans and T equinum from humans and horses. Mycopathologia..

[7] Amer AA, Tana M, Diab NA, el Moughith A, el Harras M (1993). Ultrastructure of Trichophyton violaceum. Int J Dermatol.

[8] Naka W, Fukuda T, Ohmi T, Kanai K, Nishikawa T (1995). Ultrastructure of Trichophyton mentagrophytes stained with neutral red. J Med Vet Mycol.

[9] Savitskaya TI, Vasilyeva, NV, Martynov AA, Stepanova AA, Rasnatovskiy KI (2007). Electron-microscopic investigations grooving in vitro cells of Trichophyton rubrum (Castell). Semon Probl Med Mycol.

[10] Stepanova AA (2010). Ultrastructure of the cells of Trichophyton violaceum Sabour. Probl Med Mycol.

[11] Yue X, Li Q, Wang H, Sun Y, Wang A, Zhang Q (2015). An ultrastructural study of Trichophyton rubrum induced onychomycosis. BMC Infect Dis..

[12] Pock-Steen B, Kobayasi T (1970). Ultrastructure of the hyphal wall and septum of Trichophyton mentagrophytes. J Invest Dermatol.

[13] Степанова АА, Синицкая ИА (2004). Ультраструктура Trichophyton mentagrophytes var interdigitale Blanchard. Probl Med Mycol.

[14] Sagar K (2008). Structural alterations in plant compound treated Trichophyton tonsurans. The Internet J Microbiol..

[15] Mochizuki T, Anzawa K, Sakata Y, Fujihiro M (2013). Simple identification of Trichophyton tonsurans by chlamydospore-like structures produced in culture media. J Dermatol.

[16] Васильев АЕ, Камалетдинова ФИ (1988). О фиброзиновых тельцах грибных клеток. Докл АН СССР.

[17] Harder DE (1976). Electron microscopy of urediospore formation in Puccinia coronata avenae and P graminis avenae. Can J Botany.

[18] Hassan ZM, Littlefield LJ (1979). Ontogeny of the uredium of Melampsora lini. Can J Botany.

[19] Степанова АА, Синицкая ИА (2006). Цитология клеток выращенного in vitro вегетативного мицелия Aspergillus versicolor (Vuill) Tiraboshi. Probl Med Mycol.

[20] Stepanova AA, Vasilyeva NV, Zhang F, Tong D (2016). Ultrastructural investigation of growing in vitro cells of vegetative mycelium of Aspergillus candidus Link. Probl Med Mycol.

[21] Vasilyeva NV, Stepanova AA, Sinitskaya IA, Semenov VV (2005). Comparative ultrastructural investigations of Cryptococcusneoformans strains with different virulence. Probl Med Mycol.

[22] Stepanova AA, Vasilyeva NV, Yamaguchi M (2015). Electron microscopy of autopsy material from the human brain cryptococcosis and aids. Probl Med Mycol.

[23] Stepanova AA, Synitskaiya IA (2006). Ultrastructure of the cells of vegetative mycelium Aspergillus flavus Link, growing in vitro. Probl Med Mycol.

[24] Stepanova AA, de Hoog GS, Vasilyeva NV (2016). Intra- and interspecific diversity of ultrastructural markers in Scedosporium. Fung Biol.

[25] Muller WH, Montijn RC, Humbel BM, van Aelst AC, Boon EJM, van der Krift TP (1998). Structural differences between two types of basidiomycete septal pore caps. Microbiology.

